# Co-Colonization of Non-*difficile* Clostridial Species in Antibiotic-Associated Diarrhea Caused by *Clostridioides difficile*

**DOI:** 10.3390/antibiotics14040397

**Published:** 2025-04-11

**Authors:** Daniel Salas-Treviño, Samantha Flores-Treviño, Carlos Cisneros-Rendón, Cristian Valdemar Domínguez-Rivera, Adrián Camacho-Ortiz

**Affiliations:** Department of Infectious Diseases, Hospital Universitario Dr. José Eleuterio González, Universidad Autónoma de Nuevo León, Monterrey 64460, Mexico; daniel.salastr@uanl.edu.mx (D.S.-T.); samantha.florestr@uanl.edu.mx (S.F.-T.); carlos.cisnerosrn@uanl.edu.mx (C.C.-R.); valdemar.dominguezrvr@uanl.edu.mx (C.V.D.-R.)

**Keywords:** antibiotic-associated diarrhea, *Clostridioides difficile*, non-*difficile* Clostridia, *Clostridium innocuum*, *Clostridium ramosum*

## Abstract

**Background/Objectives**: Antibiotic-associated diarrhea (AAD) is a public health problem that develops in the hospital setting. The most common causative agent of AAD is *Clostridioides difficile* infection (CDI), although other non-*difficile* Clostridia (NDC) might also be present. NDC include members of the RIC group such as *Clostridium ramosum* [*T. ramosa*], *Clostridium innocuum* and *Clostridium clostridioforme* [*E. clostridioformis*]. The co-colonization of NDC and CDI in patients with AAD has not been fully analyzed. **Methods**: We compared clinical and laboratory data of patients with *C. difficile* infection (CDI) plus NDC against patients with only CDI. This study was a retrospective, case–control study. Hospitalized confirmed CDI cases were analyzed. CDI detection was performed using a 2-step diagnostic algorithm, including glutamate dehydrogenase (GDH) with toxin A/toxin B assays and molecular detection of the *tpi* gene. Stool samples were cultured and colonies morphologically compatible with any Clostridia were identified with matrix-assisted laser desorption/ionization time-of-flight mass spectrometry. Fisher’s exact test and odds ratio (OR) were calculated to determine the degree of correlation between the variables and the study groups. **Results**: In the CDI + NDC group (*n* = 7), positive culture was observed for *C. ramosum* [*T. ramosa*] (*n* = 3), *C. innocuum* (*n* = 3), and *C. butyricum* (*n* = 1). According to our results, CDI + NDC patients received more days of antibiotic therapy, took more days to reduce diarrhea, had a significant increase in the number of days to suppress diarrhea, and previous hospitalizations were more frequently reported. **Conclusions**: In conclusion, the positive culture of NDC species such as *C. innocuum* or *C. ramosum* in patients with AAD caused by CDI correlates with treatment extension and/or failure.

## 1. Introduction

Antibiotic-associated diarrhea (AAD) is a public health problem that develops mostly in the hospital setting, with an incidence of between 5 and 25% of patients, depending on the treatment administered [1]. The most common infectious entity responsible for this condition is *Clostridioides difficile* [1], although the presence of other non-*difficile* Clostridia (NDC) has been not fully analyzed in this context. NDC can refer to the members of the RIC group, which includes *Clostridium ramosum* [*T. ramosa*], *Clostridium innocuum* and *Clostridium clostridioforme [E. clostridioformis]*. Under microscopic examination, strains belonging to the RIC group can appear as Gram-positively variable and in diverse shapes. *C. ramosum* [*T. ramosa*] can be straight or helicoidal curved rods [2] whereas *C. innocuum* is observed as straight bacilli in the shape of a double spoon or with oval ends due to its spores [3].

*C. ramosum* [*T. ramosa*], recently reclassified as *Thomasclavelia ramosa* based on 16S rRNA phylogeny [4], is part of the intestinal microbiota. However, it can also become pathogenic under certain circumstances such as bacteremia or extraintestinal infections caused by trauma or intestinal perforations [5,6], which can be treated with metronidazole, amoxicillin/clavulanate, piperacillin/tazobactam or meropenem [7]. *C. innocuum* is also a commensal bacteria colonizing the gut of up to 80% of adults [8,9], which can also cause several intestinal diseases including AAD and even bacteremia [8,10]. *C. clostridioforme,* now reclassified as *Enterocloster clostridioformis* based on 16S rRNA phylogeny [11], is also a causative agent of anaerobic bacteremia [12]. Accurate identification of the *Clostridium* strains within the RIC group is important due to their resistance to several antimicrobial agents [12].

Several methods can be used to identify anaerobes, including growth characteristics, colony morphology and susceptibility to specific antibiotics. Matrix-assisted laser desorption/ionization time-of-flight (MALDI-TOF) mass spectrometry is based on the spectra obtained from conserved ribosomal proteins, allowing rapid and accurate species identification [13].

This study aimed to identify the correlation between co-colonization with NDC and CDI in AAD. Therefore, we describe the clinical characteristics and outcomes of patients with AAD caused by *C. difficile* infection plus Clostridia non-*difficile* co-colonization (CDI + NDC) matching those of patients with AAD caused only by *C. difficile* infection (CDI).

## 2. Results

### 2.1. Characteristics of CDI Patients Included

We compared the clinical and prognostic characteristics effects of patients with CDI confirmed by 2-step algorithm testing and a positive culture and those from clinically similar patients with a virtually identical diagnostic test in whom positive culture of NDC was detected. At the time of diagnosis and culture collection, the patients had not been treated with any antibiotics during their current hospital stay.

**Figure 1:**
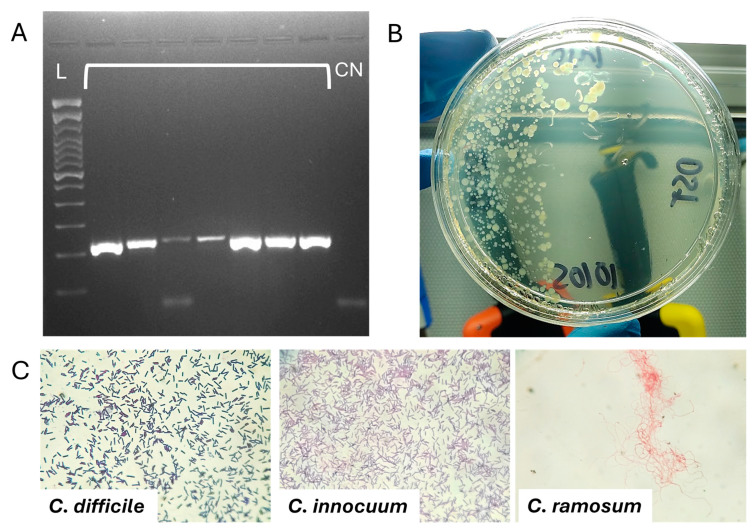
Molecular detection of *Clostridioides difficile* (CDI) and macro–microscopic morphology of non-*difficile* Clostridia (NDC). (**A**) Electrophoretic gel of the molecular detection by PCR of the *tpi* gene (230 bp) of *C. difficile*, L: DNA ladder (100 bp). Clasp lanes: samples, CN: negative control. (**B**) Macroscopic morphology of the colonies of an NDC (*Clostridium innocuum*), which are observed as small, whitish, irregular colonies with an opaque center and a light brown tone on CDA agar. (**C**) Microscopic morphology of CDI and NDC; the latter has a poor affinity to Gram and a very different morphology to CDI, being thinner bacilli, and in the case of *T. ramosa*, long and coiled. Total magnification: 400×.

The stool samples of twenty-eight patients with positive GDH were analyzed. The CDI group (*n* = 21) included patients with CDI confirmed by PCR and growth of only C. difficile in cultures. In the CDI + NDC group (*n* = 7), significant growth (50,000 CFU/mL) was observed for *T. ramosa* (*n* = 3), *C. innocuum* (*n* = 3), and *C. butyricum* (*n* = 1). Molecular detection by PCR of *C. difficile* Topoisomerase I (*tpi*) gene was positive (Figure 1A) in all samples. The macroscopic and microscopic morphologies of the colonies and bacteria are shown in Figure 1B,C.

In both groups, patients on average were 45 years of age. Both weight and body mass index were higher in the CDI group (*p* = 0.003 and 0.017, respectively) (Table 1). Likewise, the length of hospital stay, days in intensive care, ATLAS score and Charlson score (comorbidity) were higher in the CDI group.

### 2.2. Characteristics of CDI + NDC Group

The CDI + NDC group registered a greater number of stool movements compared to CDI patients, although it did not reach statistical significance (*p* = 0.096). Patients in the CDI + NDC group received more days of antibiotic treatment and significantly increased the time to cessation of diarrhea (*p* = 0.0027) compared to the CDI group (Table 1). Compared to CDI patients, leukocytosis (>16 K Cells/µL) and the detection of toxin B were less reported in the CDI + NDC patients; furthermore, all the CDI + NDC patients were previously hospitalized (*p* = 0.0302) (Table 2).

## 3. Discussion

Traditionally, *Clostridium* are anaerobic Gram-positive spore-forming bacteria. However, some species are not spore-forming, such as *T. ramosa*, some can be oxygen-tolerant, and others can be microscopically visualized as Gram-negative (*T. ramose* and *E. clostridiformis*) [13]. Therefore, based on 16S rRNA phylogeny, several species were reclassified to other genera, such as *Thomasclavelia* and *Enterocloster,* among others. Particularly, [*T. ramosa*] and *C. clostridioforme* [*E. clostridioformis*] were renamed [4,11]. Toxin-producing *C. difficile* strains can cause AAD, pseudomembranous colitis and toxic megacolon [13]. Proper treatment of clostridial infections involves the administration of antibiotic therapy, including penicillin, vancomycin or metronidazole. Thus, precise identification of the *Clostridium* strains within NDC is important due to their discrepancies among treatment options [12].

*T. ramosa* often goes unnoticed in laboratory diagnosis due to its high heterogeneity in its affinity for Gram staining and its variable colony morphology. In the last decade, *T. ramosa* reporting increased due to more reliable identification methods such as MALDI-TOF-MS [14]. To our knowledge, the presence of viable *T. ramosa* co-colonization in patients with AAD caused by CDI has not been reported, such patients showed delay in gastrointestinal stabilization and recovery. *T. ramosa* can show susceptibility to metronidazole, amoxicillin/clavulanate, piperacillin/tazobactam or meropenem, whereas resistance to penicillin, ciprofloxacin, clindamycin, imipenem and ertapenem was also reported [7].

*C. innocuum* is a frequent colonizing microorganism of the gut microbiota in adults [9]. The relative abundance of *C. innocuum* can be higher in patients exposed to antibiotics and septic patients compared with healthy controls [15]. *C. innocuum* can show susceptibility to clindamycin, metronidazole, penicillin, piperacillin and ampicillin-sulbatam but is intrinsically resistant to vancomycin and can produce biofilm [15,16]. Moreover, *tcdAB*-like genes, associated with toxins, have been identified in *C. innocuum* strains [17], exhibiting its potential ability to cause AAD in hospitalized patients. Co-colonization of *C. difficile* and *C. innocuum* is frequent in patients with AAD [18,19]. Other studies rule out such an association [20]; however, all studies adopted a molecular detection approach and none of them analyzed the viability of these species in culture. In our study, we found that patients with positive *C. innocuum* cultures were associated with longer duration of treatments to cessation of diarrhea. Other studies have also reported *C. innocuum* in patients with intestinal diseases and diarrhea, which could explain the duration of treatments to stabilize diarrhea in our patients with co-colonization [18].

*C. clostridioforme*, also a member of the RIC group, generally show resistance to beta-lactams, glycopeptides, macrolides, chloramphenicol, lincosamides, rifampin, linezolid, bacitracin, aminoglycosides and tetracyclines. These gut commensals can act as a reservoir of antimicrobial resistances, mainly conferring vancomycin resistance [12,21]. In our study, we did not detect any strain identified as *C. clostridioforme.* Nevertheless, it is important to consider the colonization with this microorganism and its antimicrobial susceptibilities when investigating AAD causative agents.

*C. butyricum*, also part of the intestinal microbiota in almost 20% of adults, shows a protective gastrointestinal role against CDI [22,23]. *C. butyricum* can be used as a probiotic to stabilize dysbiosis due to the production of short-chain fatty acids (SCFAs) [24]; however, some strains and nutritional conditions (e.g., lactose consumption) are related to pathogenic properties of this species [23]. Therefore, strain typification must be evaluated for the determination of its role in AAD. Although not part of the RIC group, one strain was detected in our study.

Gut microbiota dysbiosis also plays a role in the pathogenesis of AAD, which can be either positive or negative, depending on the relative abundance of specific species [25]. In our study, prior hospitalizations were associated with co-colonization with NDC and C. difficile. Hospitalizations are often accompanied by antibiotic treatment, which causes intestinal dysbiosis, leading to the dominance of spore-forming bacteria such as these species [1]. Therefore, it is important to consider either *C. innocuum* or *T. ramosa* infection among probable causes when the suspicion of CDI does not subside even after proper treatment. Indeed, this reason could be attributable to our patients in the NDC group, who took more days to suppress diarrhea and needed longer antibiotic treatments.

Our study has some limitations, such as the sample size. While this is a small study and requires further analysis, the association between the factors we report and consider important is significant. Also, it would be interesting to analyze the minimum inhibitory concentration values for drugs such as vancomycin/metronidazole and the biofilm formation of the NDC strains to correlate them with persistence of symptoms or treatment failure. Likewise, a more in-depth analysis of the microbiome at baseline (pre-infection) and its comparison with the expression during infection could also help define the role of NDCs and their effect on the severity of clinical conditions.

It is plausible that the presence of RIC species in patients with *Clostridioides difficile* infection (CDI) plays an active role in contributing to pathological synergy, potentially exacerbating the infection and its symptoms. Alternatively, it may simply indicate a more profound dysbiosis, a disruption in the balance of gut microbiota, particularly in patients who have undergone prior antibiotic treatments. These treatments may have disrupted the normal microbial community, allowing the overgrowth of specific species like RIC, which may not necessarily be causally linked to the pathology but rather reflect an imbalance in the gut ecosystem.

## 4. Materials and Methods

### 4.1. Study Design

This study was a retrospective, case-control age-matched study.

### 4.2. CDI Case Selection

Hospitalized adult patients with a confirmed case of CDI from January 2023 to April 2024 were analyzed. The study was conducted at the University Hospital in Monterrey, Mexico. Hospitalized patients who met the clinical case definition of 3 or more stool movements with a Bristol scale of 6 or 7 for 24 h were screened for CDI by a 2-step algorithm mentioned below and stool culture for *C. difficile*.

### 4.3. CDI Detection

The 2-step diagnostic algorithm included Glutamate dehydrogenase (GDH) and toxin A/toxin B assays (Meridian ImmunoCard Toxins A&B, Meridian Bioscience, Memphis, TN, USA) and molecular detection of *C. difficile* (*tpi* gene) through end-point PCR according to Lemee et al. [26].

All patients with a positive GDH test, at least one toxin assay positive and positive cultures with a significant growth (>50,000 CFU/mL) in anaerobic cultures of any Clostridial species were analyzed. We collect relevant clinical and laboratory data from medical records and our databases. For every CDI + NCD case, there were 3 CDI age-matched controls.

### 4.4. Ethics Statement

All procedures complied with relevant laws and institutional guidelines and have been approved by the appropriate institutional committee(s) with the approval code IF23-00001. Written informed consent was waived.

### 4.5. Stool Culture

Cultures were performed using samples used for diagnostic testing of GDH and toxins. These samples were obtained before any treatment targeting *C. difficile*. Stool samples were cultured on Reinforced Clostridial Medium EP/USP (Condalab, Madrid, Spain) and incubated under anaerobic conditions (CO_2_ 10%, H_2_ 5%, balanced with N_2_) at 37 °C for 4 days. Colonies morphologically compatible with Clostridia, i.e., irregular, raised, convex grey colonies of 4–6 mm diameter, were further selected.

### 4.6. Clostridia Species Identification

Species identification was performed in 10 fully growing colonies of each morphotype compatible with clostridia. The identification was carried out with matrix-assisted laser desorption/ionization time-of-flight mass spectrometry (MALDI-TOF MS) (Microflex LT system, Bruker Daltonics, Bremen, Germany) according to the manufacturer’s instructions, using the protein tube extraction method. The colonies were resuspended in 300 µL of water, 900 µL of ethanol was added, and the sample was centrifuged at 13,000 rpm for 2 min and the supernatant was discarded. Then, 20 µL of 70% formic acid (Fermont, Monterrey, Mexico) and 20 µL of acetonitrile (Fermont, NL, Mexico) were added. After centrifugation at 13,000 rpm for 2 min, 1 µL of the supernatant was transferred into a 96-well stainless-steel plate (Bruker Daltonics) and 1 µL of alpha-cyano-4-hydroxycinnamic acid matrix solution (Sigma Aldrich, Toluca, Mexico) was added. The plate was analyzed using the MALDI Biotyper 3.0 software to obtain spectral profiles and match them with the database. Scores above 2.00 were used as acceptable criteria for species-level identification.

### 4.7. Statistical Analysis

Quantifiable clinical data were described as means and standard deviation, and qualitative data were reported as frequencies and percentages. Comparative tests of means such as the Student’s *t*-test and the Mann–Whitney U test were performed according to the data distribution. Fisher’s exact test and odds ratio (OR) were calculated to determine the degree of correlation between the variables and the study groups. A *p*-value < 0.05 was considered as statistically significant and a value between 0.05 and 0.1 was considered as a statistical trend. The alpha error value was 5.0% in all the tests. The software SPSS^®^ version 25.0 (IBM™, New York, NY, USA) was used.

## 5. Conclusions

In this study, we observed laboratory and clinical data related to CDI + NDC compared to CDI, such as history of previous hospitalization and longer treatment days to suppress diarrhea. These clinical and laboratory values are relevant when establishing the diagnosis and treatment of AAD, especially those that do not remit with conventional treatment, as it increases the possibility of NDC co-colonization. Although the number of samples and cultures performed in this short report are limited, we consider that cultivable co-colonization of NDC such as *C. innocuum* or *T. ramose* with *C. difficile* in AAD patients can be associated with treatment failure or prolongation.

## Figures and Tables

**Table 1:** Quantitative values of clinical and demographic characteristics of the study populations.

Patient Characteristic	CDI + NDC (*n* = 7)	CDI (*n* = 21)	*p*-Value
Age (years)	45.57 ± 23.02	45.33 ± 22.06	**0.9807 ^a^**
Patient weight (kg)	59.16 ± 9.55	72.38 ± 9.17	**0.0030 ^a^**
BMI	22.48 ± 4.21	26.41 ± 3.31	**0.0179 ^a^**
Hospital LOS	25.57 ± 17.90	32.00 ± 26.64	0.5592 **^a^**
Charlson Score	2.85 ± 2.03	3.23 ± 3.46	0.7584 ^b^
LOS in ICU	0.83 ± 1.32	2.04 ± 6.02	0.7250 ^b^
Bowel movements per day	6.71 ± 2.81	4.90 ± 2.24	0.0967 **^a^**
Total leukocytes (Cel/µL)	11.07 ± 4.05	12.11 ± 7.52	0.7324 **^a^**
Albumin (g/dL)	2.11 ± 0.51	2.35 ± 0.77	0.6497 ^b^
Creatinine (mg/dL)	1.47 ± 1.51	3.47 ± 6.27	0.6027 ^b^
ATLAS score	3.28 ± 0.75	4.14 ± 1.59	0.1846 **^a^**
Days of antibiotic treatment	11.14 ±1.95	8.65 ± 4.69	0.1888 **^a^**
Time to cessation of diarrhea	5.57 ± 1.81	3.13 ± 0.65	**0.0027 ^a^**

^a^ Unpaired *t*-test; ^b^ Mann–Whitney U test. Bold *p*-values represent statistical significance. LOS: length of stay (days). Cel/µL: cells per microliter of whole blood, kg: kilograms, g/dL: grams per deciliter of serum, mg/dL: milligrams per deciliter of serum. ATLAS: age, treatment with systemic antibiotics, leukocyte count, albumin, and serum creatinine; CDI: *Clostridioides difficile* infection; NDC: non-*difficile* Clostridia; ICU: intensive care unit.

**Table 2:** Frequency analysis of clinical variables of the study population.

Patient Characteristic	CDI + NDC (*n*, %)	CDI (*n*, %)	OR (CI)	*p*-Value
Toxin A detectable	6 (85.7)	18 (100)	0.11 (0.004–3.25)	0.2800
Toxin B detectable	4 (57.1)	16 (88.9)	0.16 (0.02–1.35)	0.1130
PPIs	6 (85.7)	12 (57.1)	4.50 (0.45–44.31)	0.3642
Leukocytes >16 K Cel/µL	0 (0.0)	8 (38.1)	0.10 (0.005–2.10)	0.0749
Treatment with Metronidazole/Vancomycin	3 (42.9)	4 (19.0)	3.18 (0.50–20.31)	0.3183
Treatment with Vancomycin	5 (71.4)	19 (90.5)	0.26 (0.02–2.36)	0.2530
Antibiotic switch treatment	2 (28.6)	1 (5.6)	6.80 (0.50–91.55)	0.1796
Previous Hospitalization	7 (100)	11 (52.4)	13.70 (0.69–270.5)	**0.0302**
Hospitalization in ICU	3 (42.9)	4 (19.0)	3.18 (0.50–20.31)	0.3183
Attributable mortality to CDI	0 (0.0)	3 (14.3)	0.35 (0.01–7.69)	0.5513

Fisher’s exact test. OR: odds ratio. CI: confidence interval. PPIs: proton-pump inhibitors, Cel/µL: cells per microliter of whole blood, ICU: intensive care unit, CDI: *Clostridioides difficile* infection, NDC: non-*difficile* Clostridia. Bold letters indicate statistical significance *p <* 0.05.

## Data Availability

Data used are contained within the article and Supplementary Material.

## Supplementary Materials

The following supporting information can be downloaded at: https://www.mdpi.com/article/10.3390/antibiotics14040397/s1, The results of the identification of the Non-*difficile* Clostridia isolates by MALDI-TOF used in this study.
