# Supplementary material for: Co-Colonization of Non-difficile Clostridial Species in Antibiotic-Associated Diarrhea Caused by Clostridioides difficile
Source: Antibiotics (Basel). 2025 Apr 11;14(4):397. doi: 10.3390/antibiotics14040397 (PMC12024451; doi:10.3390/antibiotics14040397)
Supplement: Supplementary file 1 [file antibiotics-14-00397-s001.zip › antibiotics-3531785-supplementary.pdf]

Bruker MALDI Biotyper Identification Results

Run Info:

**Run Identifier:**	230825-1502-1011008527
**Comment:**	
**Operator:**	Admin@MBT-WIN10
**Run Creation Date/Time:**	2023-08-25T15:03:39.178
**Number of Tests:**	4
**Type:**	Standard
**BTS-QC:**	not present
**BTS-QC Position:**	
**Instrument ID:**	604674.01802
**Server Version:**	4.1.100 (PYTH) 174 2019-06-158_01-16-09

Result Overview

Sample Name	Sample ID	Organism (best match)	Score Value	Organism (second-best match)	Score Value
A10 (+++) (A)	2023-17.1 (Standard)	Clostridioides difficile	2.05	Clostridioides difficile	1.93
A11 (+) (B)	2023-17.2 (Standard)	Clostridioides difficile	1.96	Clostridioides difficile	1.89
A12 (+++) (A)	2023-18.1 (Standard)	Clostridium ramosum	2.42	Clostridium ramosum	2.27
B1 (-) (C)	2023-18.2 (Standard)	**no peaks found**	0.00	**no peaks found**	0.00

Matching Hints

Matched Pattern	Comment
Clostridioides difficile 0422_0288_DM IBS	synonym of Clostridium difficile
Clostridioides difficile DSM 12057 DSM	synonym of Clostridium difficile
Clostridioides difficile DSM 1296T DSM	synonym of Clostridium difficile
Clostridioides difficile MB_1562_05 THL	synonym of Clostridium difficile
Clostridioides difficile MB_4499_05 THL	synonym of Clostridium difficile
Clostridioides difficile MB_7476_05 THL	synonym of Clostridium difficile

Meaning of Score Values

Range	Interpretation	Symbols	Color
2.00 - 3.00	High-confidence identification	(+++)	green
1.70 - 1.99	Low-confidence identification	(+)	yellow
0.00 - 1.69	No Organism Identification Possible	(-)	red

Meaning of Consistency Categories (A - C)

Category	Interpretation
(A)	**High consistency:** The best match is a high-confidence identification. The second-best match is (1) a high-confidence identification in which the species is identical to the best match, (2) a low-confidence identification in which the species or genus is identical to the best match, or (3) a non-identification.
(B)	**Low consistency:** The requirements for high consistency are not met. The best match is a high- or low-confidence identification. The second-best match is (1) a high- or low-confidence identification in which the genus is identical to the best match or (2) a non-identification.
(C)	**No consistency:** The requirements for high or low consistency are not met.

Sample 1

**Sample Name:**	A10
**Sample Description:**	
**Sample ID:**	2023-17.1
**Sample Creation Date/Time:**	2023-08-25T15:03:39.222
**Sample Type:**	Standard
**Identification Method:**	MALDI Biotyper MSP Identification Standard Method_MIXED 1.1
**Preprocessing Method:**	MALDI Biotyper Preprocessing Standard Method 1.1
**ACQ Method:**	D:\Methods\flexControlMethods\MBT_FC.par
**AutoXecute Method:**	MBT_AutoX
**Consistency Category (based on 2 best matches):**	A
**Applied Taxonomy Tree:**	Bruker Taxonomy

Rank (Quality)	Matched Pattern	Score Value	NCBI Identifier
1 (+++)	Clostridioides difficile DSM 1296T DSM	2.05	1496
2 (+)	Clostridioides difficile 0422_0288_DM IBS	1.93	1496
3 (+)	Clostridioides difficile DSM 12057 DSM	1.90	1496
4 (-)	Terrisporobacter glycolicus DSM 1288T DSM	1.54	186804
5 (-)	Terrisporobacter glycolicus DSM 13865 DSM	1.41	186804
6 (-)	Peptostreptococcus canis 3134_15 HITG	1.39	1257
7 (-)	Alcaligenes faecalis ssp faecalis DSM 30030T HAM	1.36	511
8 (-)	Methylobacillus sp MB118 UFL	1.27	404
9 (-)	Terrisporobacter glycolicus 203246 RLH	1.23	36841
10 (-)	Staphylococcus aureus ATCC 33591 THL	1.17	1280

Sample 2

**Sample Name:**	A11
**Sample Description:**	
**Sample ID:**	2023-17.2
**Sample Creation Date/Time:**	2023-08-25T15:03:39.224
**Sample Type:**	Standard
**Identification Method:**	MALDI Biotyper MSP Identification Standard Method_MIXED 1.1
**Preprocessing Method:**	MALDI Biotyper Preprocessing Standard Method 1.1
**ACQ Method:**	D:\Methods\flexControlMethods\MBT_FC.par
**AutoXecute Method:**	MBT_AutoX
**Consistency Category (based on 2 best matches):**	B
**Applied Taxonomy Tree:**	Bruker Taxonomy

Rank (Quality)	Matched Pattern	Score Value	NCBI Identifier
1 (+)	Clostridioides difficile DSM 1296T DSM	1.96	1496
2 (+)	Clostridioides difficile DSM 12057 DSM	1.89	1496
3 (+)	Clostridioides difficile MB_4499_05 THL	1.84	1496
4 (+)	Clostridioides difficile 0422_0288_DM IBS	1.75	1496
5 (-)	Clostridioides difficile MB_1562_05 THL	1.40	1496
6 (-)	Clostridioides difficile MB_7476_05 THL	1.37	1496
7 (-)	Staphylococcus succinus ssp succinus DSM 14617T DSM	1.34	61015
8 (-)	Peptostreptococcus canis 3134_15 HITG	1.28	1257
9 (-)	Terrisporobacter glycolicus DSM 1288T DSM	1.26	186804
10 (-)	Bacillus clarkii DSM 8720T DSM	1.24	-

Sample 3

**Sample Name:**	A12
**Sample Description:**	
**Sample ID:**	2023-18.1
**Sample Creation Date/Time:**	2023-08-25T15:03:39.226
**Sample Type:**	Standard
**Identification Method:**	MALDI Biotyper MSP Identification Standard Method_MIXED 1.1
**Preprocessing Method:**	MALDI Biotyper Preprocessing Standard Method 1.1
**ACQ Method:**	D:\Methods\flexControlMethods\MBT_FC.par
**AutoXecute Method:**	MBT_AutoX
**Consistency Category (based on 2 best matches):**	A
**Applied Taxonomy Tree:**	Bruker Taxonomy

Rank (Quality)	Matched Pattern	Score Value	NCBI Identifier
1 (+++)	Clostridium ramosum 14134972_4 MVD	2.42	1547
2 (+++)	Clostridium ramosum 14138811_8 MVD	2.27	1547
3 (+++)	Clostridium ramosum 1C15007811_8 MVD	2.25	1547
4 (+++)	Clostridium ramosum 94 PIM	2.18	1547
5 (+++)	Clostridium ramosum 470 RLT	2.17	1547
6 (+++)	Clostridium ramosum IBS_MS_15 IBS	2.06	1547
7 (+++)	Clostridium ramosum CCUG 45030 CCUG	2.06	1547
8 (+++)	Clostridium ramosum 15_758 IBS	2.00	1547
9 (+)	Clostridium ramosum DSM 1402T VML	1.92	1547
10 (-)	Clostridium ramosum 49 RLT	1.69	1547

Sample 4

**Sample Name:**	B1
**Sample Description:**	
**Sample ID:**	2023-18.2
**Sample Creation Date/Time:**	2023-08-25T15:03:39.228
**Sample Type:**	Standard
**Identification Method:**	MALDI Biotyper MSP Identification Standard Method_MIXED 1.1
**Preprocessing Method:**	MALDI Biotyper Preprocessing Standard Method 1.1
**ACQ Method:**	D:\Methods\flexControlMethods\MBT_FC.par
**AutoXecute Method:**	MBT_AutoX
**Consistency Category (based on 2 best matches):**	C
**Applied Taxonomy Tree:**	Bruker Taxonomy

Rank (Quality)	Matched Pattern	Score Value	NCBI Identifier
1 (-)	**no peaks found**	0.00	-
2 (-)	**no peaks found**	0.00	-
3 (-)	**no peaks found**	0.00	-
4 (-)	**no peaks found**	0.00	-
5 (-)	**no peaks found**	0.00	-
6 (-)	**no peaks found**	0.00	-
7 (-)	**no peaks found**	0.00	-
8 (-)	**no peaks found**	0.00	-
9 (-)	**no peaks found**	0.00	-
10 (-)	**no peaks found**	0.00	-

Bruker MALDI Biotyper Identification Results

Run Info:

**Run Identifier:**	250311-1549-1011008527
**Comment:**	
**Operator:**	Admin@MBT-WIN10
**Run Creation Date/Time:**	2025-03-11T15:59:50.971
**Number of Tests:**	10
**Type:**	Standard
**BTS-QC:**	not present
**BTS-QC Position:**	
**Instrument ID:**	604674.01802
**Server Version:**	4.1.100 (PYTH) 174 2019-06-158_01-16-09

Result Overview

Sample Name	Sample ID	Organism (best match)	Score Value	Organism (second-best match)	Score Value
G4 (+++) (A)	Ma GPE (Standard)	Clostridium butyricum	2.12	Clostridium butyricum	2.10
G5 (+++) (A)	Karla (Standard)	Clostridium perfringens	2.43	Clostridium perfringens	2.33
G6 (+++) (A)	Ma GPE (Standard)	Clostridium butyricum	2.24	Clostridium butyricum	2.22
G7 (+++) (A)	Karla (Standard)	Clostridium perfringens	2.32	Clostridium perfringens	2.30
G8 (+++) (A)	183 (Standard)	Klebsiella pneumoniae	2.05	Klebsiella pneumoniae	1.94
G9 (-) (A)	183 (Standard)	**No Organism Identification Possible**	1.61	**No Organism Identification Possible**	1.60
G10 (+++) (A)	2386 (Standard)	Pseudomonas aeruginosa	2.26	Pseudomonas aeruginosa	2.13

Result overview table--continued on next page

Result overview table--continued from previous page					
**Sample Name**	**Sample ID**	**Organism (best match)**	**Score Value**	**Organism (second-best match)**	**Score Value**
G11 (-) (A)	2386 (Standard)	**no peaks found**	0.00	**no peaks found**	0.00
G12 (-) (C)	262 (Standard)	**no peaks found**	0.00	**no peaks found**	0.00
H1 (-) (C)	262 (Standard)	**no peaks found**	0.00	**no peaks found**	0.00

Matching Hints

Matched Pattern	Comment
Clostridioides difficile 0422_0288_DM IBS	synonym of Clostridium difficile
Clostridioides difficile DSM 1296T DSM	synonym of Clostridium difficile
Haemophilus parahaemolyticus CIP 100082 CIP	Species parahaemolyticus / paraphrohaemolyticus of the genus Haemophilus have very similar patterns: Therefore distinguishing their species is difficult.
Haemophilus parahaemolyticus VP 58527 BOM	Species parahaemolyticus / paraphrohaemolyticus of the genus Haemophilus have very similar patterns: Therefore distinguishing their species is difficult.
Klebsiella aerogenes 15282_1 CHB	synonym of Enterobacter aerogenes
Klebsiella aerogenes ATCC 13048T THL	synonym of Enterobacter aerogenes
Klebsiella pneumoniae 37585 PFM	closely related to Klebsiella variicola
Klebsiella pneumoniae RV_BA_03_B LBK	closely related to Klebsiella variicola
Klebsiella pneumoniae ssp ozaenae DSM 16358T HAM	closely related to Klebsiella variicola
Klebsiella pneumoniae ssp pneumoniae 9295_1 CHB	closely related to Klebsiella variicola
Klebsiella pneumoniae ssp pneumoniae DSM 30104T HAM	closely related to Klebsiella variicola
Klebsiella pneumoniae ssp pneumoniae DSM 30104T_QC DSM	closely related to Klebsiella variicola
Klebsiella pneumoniae ssp rhinoscleromatis DSM 16231T HAM	closely related to Klebsiella variicola
Klebsiella variicola DSM 15968T DSM	closely related to Klebsiella pneumoniae

Matching Hints table--continued on next page

Matching Hints table--continued from previous page	
**Matched Pattern**	**Comment**
Paenarthrobacter aurescens DSM 20116T DSM	The taxonomical species diversity within the genus Paenarthrobacter is very high. Many species are closely related to each other and build groups or clusters. Such species cluster could consist of up to 15 different species and can be even overlapping with other clusters. The given MALDI Biotyper identification shall be generally interpreted as a preliminary result. For exact species identification further differentiating methods have to be selected by an experienced professional. Please consider and expect a similar limited species resolution by using 16S rRNA gene sequencing.
Secundilactobacillus malefermentans DSM 5705T DSM	synonym of Lactobacillus malefermentans

Meaning of Score Values

Range	Interpretation	Symbols	Color
2.00 - 3.00	High-confidence identification	(+++)	green
1.70 - 1.99	Low-confidence identification	(+)	yellow
0.00 - 1.69	No Organism Identification Possible	(-)	red

Meaning of Consistency Categories (A - C)

Category	Interpretation
(A)	**High consistency:** The best match is a high-confidence identification. The second-best match is (1) a high-confidence identification in which the species is identical to the best match, (2) a low-confidence identification in which the species or genus is identical to the best match, or (3) a non-identification.
(B)	**Low consistency:** The requirements for high consistency are not met. The best match is a high- or low-confidence identification. The second-best match is (1) a high- or low-confidence identification in which the genus is identical to the best match or (2) a non-identification.
(C)	**No consistency:** The requirements for high or low consistency are not met.

Sample 1

**Sample Name:**	G4
**Sample Description:**	
**Sample ID:**	Ma GPE
**Sample Creation Date/Time:**	2025-03-11T15:51:44.961
**Sample Type:**	Standard
**Identification Method:**	MALDI Biotyper MSP Identification Standard Method_MIXED 1.1
**Preprocessing Method:**	MALDI Biotyper Preprocessing Standard Method 1.1
**ACQ Method:**	D:\Methods\flexControlMethods\MBT_FC.par
**AutoXecute Method:**	MBT_AutoX
**Consistency Category (based on 2 best matches):**	A
**Applied MSP Library(ies):**	BDAL / contains 12438 MSPs / 65db91b4-8840-4168-9c41-623f3efc8441 / 2024-10-22T23:59:27.714

Rank (Quality)	Matched Pattern	Score Value	NCBI Identifier
1 (+++)	Clostridium butyricum 0041_13_003_01_2 THH	2.12	1492
2 (+++)	Clostridium butyricum 21951 RLH	2.10	1492
3 (+++)	Clostridium butyricum 0040_13_003_01_4 THH	2.04	1492
4 (+)	Clostridium butyricum 0041_12_009_01_2 THH	1.92	1492
5 (+)	Clostridium butyricum DSM 10702T DSM	1.84	1492
6 (+)	Clostridium butyricum 900200369 LBK	1.78	1492
7 (+)	Clostridium butyricum DSM 2478 DSM	1.75	1492
8 (+)	Clostridium butyricum DSM 2477 DSM	1.73	1492
9 (-)	Clostridium butyricum 0031_13_010_01_2 THH	1.68	1492
10 (-)	Clostridioides difficile 0422_0288_DM IBS	1.63	1496

Sample 2

**Sample Name:**	G5
**Sample Description:**	
**Sample ID:**	Karla
**Sample Creation Date/Time:**	2025-03-11T15:51:44.963
**Sample Type:**	Standard
**Identification Method:**	MALDI Biotyper MSP Identification Standard Method_MIXED 1.1
**Preprocessing Method:**	MALDI Biotyper Preprocessing Standard Method 1.1
**ACQ Method:**	D:\Methods\flexControlMethods\MBT_FC.par
**AutoXecute Method:**	MBT_AutoX
**Consistency Category (based on 2 best matches):**	A
**Applied MSP Library(ies):**	BDAL / contains 12438 MSPs / 65db91b4-8840-4168-9c41-623f3efc8441 / 2024-10-22T23:59:27.714

Rank (Quality)	Matched Pattern	Score Value	NCBI Identifier
1 (+++)	Clostridium perfringens RV_BA_03_D LBK	2.43	1502
2 (+++)	Clostridium perfringens DSM 628 VML	2.33	1502
3 (+++)	Clostridium perfringens HU51221 PNU	2.30	1502
4 (+++)	Clostridium perfringens DSM 756T VML	2.23	1502
5 (+++)	Clostridium perfringens HU65618 PNU	2.20	1502
6 (+++)	Clostridium perfringens DSM 11784 VML	2.19	1502
7 (+++)	Clostridium perfringens DSM 11778_3d VML	2.18	1502
8 (+++)	Clostridium perfringens DSM 798 VML	2.14	1502
9 (+)	Clostridium perfringens DSM 11781 VML	1.87	1502
10 (+)	Clostridium perfringens ENR_0383 ENR	1.74	1502

Sample 3

**Sample Name:**	G6
**Sample Description:**	
**Sample ID:**	Ma GPE
**Sample Creation Date/Time:**	2025-03-11T15:51:44.965
**Sample Type:**	Standard
**Identification Method:**	MALDI Biotyper MSP Identification Standard Method_MIXED 1.1
**Preprocessing Method:**	MALDI Biotyper Preprocessing Standard Method 1.1
**ACQ Method:**	D:\Methods\flexControlMethods\MBT_FC.par
**AutoXecute Method:**	MBT_AutoX
**Consistency Category (based on 2 best matches):**	A
**Applied MSP Library(ies):**	BDAL / contains 12438 MSPs / 65db91b4-8840-4168-9c41-623f3efc8441 / 2024-10-22T23:59:27.714

Rank (Quality)	Matched Pattern	Score Value	NCBI Identifier
1 (+++)	Clostridium butyricum 0041_13_003_01_2 THH	2.24	1492
2 (+++)	Clostridium butyricum 21951 RLH	2.22	1492
3 (+++)	Clostridium butyricum 0040_13_003_01_4 THH	2.13	1492
4 (+++)	Clostridium butyricum 0041_12_009_01_2 THH	2.05	1492
5 (+)	Clostridium butyricum DSM 2477 DSM	1.98	1492
6 (+)	Clostridium butyricum 900200369 LBK	1.92	1492
7 (+)	Clostridium butyricum DSM 10702T DSM	1.88	1492
8 (+)	Clostridium butyricum DSM 2478 DSM	1.85	1492
9 (+)	Clostridium butyricum 0031_13_010_01_2 THH	1.72	1492
10 (-)	Clostridioides difficile DSM 1296T DSM	1.53	1496

Sample 4

**Sample Name:**	G7
**Sample Description:**	
**Sample ID:**	Karla
**Sample Creation Date/Time:**	2025-03-11T15:51:44.967
**Sample Type:**	Standard
**Identification Method:**	MALDI Biotyper MSP Identification Standard Method_MIXED 1.1
**Preprocessing Method:**	MALDI Biotyper Preprocessing Standard Method 1.1
**ACQ Method:**	D:\Methods\flexControlMethods\MBT_FC.par
**AutoXecute Method:**	MBT_AutoX
**Consistency Category (based on 2 best matches):**	A
**Applied MSP Library(ies):**	BDAL / contains 12438 MSPs / 65db91b4-8840-4168-9c41-623f3efc8441 / 2024-10-22T23:59:27.714

Rank (Quality)	Matched Pattern	Score Value	NCBI Identifier
1 (+++)	Clostridium perfringens RV_BA_03_D LBK	2.32	1502
2 (+++)	Clostridium perfringens DSM 628 VML	2.30	1502
3 (+++)	Clostridium perfringens DSM 756T VML	2.26	1502
4 (+++)	Clostridium perfringens HU51221 PNU	2.23	1502
5 (+++)	Clostridium perfringens DSM 11784 VML	2.22	1502
6 (+++)	Clostridium perfringens DSM 11778_3d VML	2.17	1502
7 (+++)	Clostridium perfringens HU65618 PNU	2.16	1502
8 (+++)	Clostridium perfringens DSM 798 VML	2.14	1502
9 (+)	Clostridium perfringens DSM 11781 VML	1.93	1502
10 (+)	Clostridium perfringens ENR_0383 ENR	1.77	1502

Sample 5

**Sample Name:**	G8
**Sample Description:**	
**Sample ID:**	183
**Sample Creation Date/Time:**	2025-03-11T15:51:44.968
**Sample Type:**	Standard
**Identification Method:**	MALDI Biotyper MSP Identification Standard Method_MIXED 1.1
**Preprocessing Method:**	MALDI Biotyper Preprocessing Standard Method 1.1
**ACQ Method:**	D:\Methods\flexControlMethods\MBT_FC.par
**AutoXecute Method:**	MBT_AutoX
**Consistency Category (based on 2 best matches):**	A
**Applied MSP Library(ies):**	BDAL / contains 12438 MSPs / 65db91b4-8840-4168-9c41-623f3efc8441 / 2024-10-22T23:59:27.714

Rank (Quality)	Matched Pattern	Score Value	NCBI Identifier
1 (+++)	Klebsiella pneumoniae ssp pneumoniae 9295_1 CHB	2.05	573
2 (+)	Klebsiella pneumoniae ssp pneumoniae DSM 30104T_QC DSM	1.94	573
3 (+)	Klebsiella pneumoniae ssp pneumoniae DSM 30104T HAM	1.83	573
4 (+)	Klebsiella aerogenes 15282_1 CHB	1.82	28451
5 (+)	Klebsiella pneumoniae 37585 PFM	1.79	573
6 (+)	Klebsiella pneumoniae ssp ozaenae DSM 16358T HAM	1.77	573
7 (+)	Klebsiella pneumoniae RV_BA_03_B LBK	1.76	573
8 (+)	Klebsiella aerogenes ATCC 13048T THL	1.76	28451
9 (-)	Klebsiella pneumoniae ssp rhinoscleromatis DSM 16231T HAM	1.67	573
10 (-)	Klebsiella variicola DSM 15968T DSM	1.61	244366

Sample 6

**Sample Name:**	G9
**Sample Description:**	
**Sample ID:**	183
**Sample Creation Date/Time:**	2025-03-11T15:51:44.970
**Sample Type:**	Standard
**Identification Method:**	MALDI Biotyper MSP Identification Standard Method_MIXED 1.1
**Preprocessing Method:**	MALDI Biotyper Preprocessing Standard Method 1.1
**ACQ Method:**	D:\Methods\flexControlMethods\MBT_FC.par
**AutoXecute Method:**	MBT_AutoX
**Consistency Category (based on 2 best matches):**	A
**Applied MSP Library(ies):**	BDAL / contains 12438 MSPs / 65db91b4-8840-4168-9c41-623f3efc8441 / 2024-10-22T23:59:27.714

Rank (Quality)	Matched Pattern	Score Value	NCBI Identifier
1 (-)	Terrisporobacter glycolicus 100617_23 PNU	1.61	36841
2 (-)	Terrisporobacter glycolicus 203246 RLH	1.60	36841
3 (-)	Secundilactobacillus malefermentans DSM 5705T DSM	1.41	176292
4 (-)	Haemophilus parahaemolyticus VP 58527 BOM	1.36	735
5 (-)	Staphylococcus succinus ssp succinus DSM 14617T DSM	1.35	61015
6 (-)	Lactobacillus curvatus DSM 20019T DSM	1.34	28038
7 (-)	Terrisporobacter glycolicus DSM 1288T DSM	1.33	186804
8 (-)	Paenarthrobacter aurescens DSM 20116T DSM	1.32	43663
9 (-)	Lactobacillus paralimentarius DSM 13238T DSM	1.29	83526
10 (-)	Haemophilus parahaemolyticus CIP 100082 CIP	1.29	735

Sample 7

**Sample Name:**	G10
**Sample Description:**	
**Sample ID:**	2386
**Sample Creation Date/Time:**	2025-03-11T15:51:44.971
**Sample Type:**	Standard
**Identification Method:**	MALDI Biotyper MSP Identification Standard Method_MIXED 1.1
**Preprocessing Method:**	MALDI Biotyper Preprocessing Standard Method 1.1
**ACQ Method:**	D:\Methods\flexControlMethods\MBT_FC.par
**AutoXecute Method:**	MBT_AutoX
**Consistency Category (based on 2 best matches):**	A
**Applied MSP Library(ies):**	BDAL / contains 12438 MSPs / 65db91b4-8840-4168-9c41-623f3efc8441 / 2024-10-22T23:59:27.714

Rank (Quality)	Matched Pattern	Score Value	NCBI Identifier
1 (+++)	Pseudomonas aeruginosa ATCC 27853 THL	2.26	287
2 (+++)	Pseudomonas aeruginosa 8147_2 CHB	2.13	287
3 (+++)	Pseudomonas aeruginosa DSM 1117 DSM	2.13	287
4 (+)	Pseudomonas aeruginosa DSM 50071T_QC DSM	1.99	287
5 (+)	Pseudomonas aeruginosa A07_08_Pudu FLR	1.98	287
6 (+)	Pseudomonas aeruginosa DSM 50071T HAM	1.97	287
7 (+)	Pseudomonas aeruginosa 19955_1 CHB	1.96	287
8 (+)	Pseudomonas aeruginosa DSM 1128 DSM	1.74	287
9 (+)	Pseudomonas aeruginosa LMG 8029 LMG	1.72	287
10 (-)	Pseudomonas jinjuensis LMG 21316T HAM	1.57	198616

Sample 8

**Sample Name:**	G11
**Sample Description:**	
**Sample ID:**	2386
**Sample Creation Date/Time:**	2025-03-11T15:51:44.973
**Sample Type:**	Standard
**Identification Method:**	MALDI Biotyper MSP Identification Standard Method_MIXED 1.1
**Preprocessing Method:**	MALDI Biotyper Preprocessing Standard Method 1.1
**ACQ Method:**	D:\Methods\flexControlMethods\MBT_FC.par
**AutoXecute Method:**	MBT_AutoX
**Consistency Category (based on 2 best matches):**	A
**Applied MSP Library(ies):**	BDAL / contains 12438 MSPs / 65db91b4-8840-4168-9c41-623f3efc8441 / 2024-10-22T23:59:27.714

Rank (Quality)	Matched Pattern	Score Value	NCBI Identifier
1 (-)	**no peaks found**	0.00	-
2 (-)	**no peaks found**	0.00	-
3 (-)	**no peaks found**	0.00	-
4 (-)	**no peaks found**	0.00	-
5 (-)	**no peaks found**	0.00	-
6 (-)	**no peaks found**	0.00	-
7 (-)	**no peaks found**	0.00	-
8 (-)	**no peaks found**	0.00	-
9 (-)	**no peaks found**	0.00	-
10 (-)	**no peaks found**	0.00	-

Sample 9

**Sample Name:**	G12
**Sample Description:**	
**Sample ID:**	262
**Sample Creation Date/Time:**	2025-03-11T15:51:44.975
**Sample Type:**	Standard
**Identification Method:**	MALDI Biotyper MSP Identification Standard Method_MIXED 1.1
**Preprocessing Method:**	MALDI Biotyper Preprocessing Standard Method 1.1
**ACQ Method:**	D:\Methods\flexControlMethods\MBT_FC.par
**AutoXecute Method:**	MBT_AutoX
**Consistency Category (based on 2 best matches):**	C
**Applied MSP Library(ies):**	BDAL / contains 12438 MSPs / 65db91b4-8840-4168-9c41-623f3efc8441 / 2024-10-22T23:59:27.714

Rank (Quality)	Matched Pattern	Score Value	NCBI Identifier
1 (-)	**no peaks found**	0.00	-
2 (-)	**no peaks found**	0.00	-
3 (-)	**no peaks found**	0.00	-
4 (-)	**no peaks found**	0.00	-
5 (-)	**no peaks found**	0.00	-
6 (-)	**no peaks found**	0.00	-
7 (-)	**no peaks found**	0.00	-
8 (-)	**no peaks found**	0.00	-
9 (-)	**no peaks found**	0.00	-
10 (-)	**no peaks found**	0.00	-

Sample 10

**Sample Name:**	H1
**Sample Description:**	
**Sample ID:**	262
**Sample Creation Date/Time:**	2025-03-11T15:51:44.976
**Sample Type:**	Standard
**Identification Method:**	MALDI Biotyper MSP Identification Standard Method_MIXED 1.1
**Preprocessing Method:**	MALDI Biotyper Preprocessing Standard Method 1.1
**ACQ Method:**	D:\Methods\flexControlMethods\MBT_FC.par
**AutoXecute Method:**	MBT_AutoX
**Consistency Category (based on 2 best matches):**	C
**Applied MSP Library(ies):**	BDAL / contains 12438 MSPs / 65db91b4-8840-4168-9c41-623f3efc8441 / 2024-10-22T23:59:27.714

Rank (Quality)	Matched Pattern	Score Value	NCBI Identifier
1 (-)	**no peaks found**	0.00	-
2 (-)	**no peaks found**	0.00	-
3 (-)	**no peaks found**	0.00	-
4 (-)	**no peaks found**	0.00	-
5 (-)	**no peaks found**	0.00	-
6 (-)	**no peaks found**	0.00	-
7 (-)	**no peaks found**	0.00	-
8 (-)	**no peaks found**	0.00	-
9 (-)	**no peaks found**	0.00	-
10 (-)	**no peaks found**	0.00	-

Bruker MALDI Biotyper Identification Results

Run Info:

**Run Identifier:**	231010-1543-1011008527
**Comment:**	
**Operator:**	Admin@MBT-WIN10
**Run Creation Date/Time:**	2023-10-10T15:43:49.756
**Number of Tests:**	2
**Type:**	Standard
**BTS-QC:**	not present
**BTS-QC Position:**	
**Instrument ID:**	604674.01802
**Server Version:**	4.1.100 (PYTH) 174 2019-06-158_01-16-09

Result Overview

Sample Name	Sample ID	Organism (best match)	Score Value	Organism (second-best match)	Score Value
F1 (+++) (A)	2023-42.1 (Standard)	Clostridium innocuum	2.24	Clostridium innocuum	2.18
F2 (+++) (A)	2023-42.2 (Standard)	Clostridium innocuum	2.23	Clostridium innocuum	2.12

Matching Hints

Matched Pattern	Comment

Meaning of Score Values

Range	Interpretation	Symbols	Color
2.00 - 3.00	High-confidence identification	(+++)	green
1.70 - 1.99	Low-confidence identification	(+)	yellow
0.00 - 1.69	No Organism Identification Possible	(-)	red

Meaning of Consistency Categories (A - C)

Category	Interpretation
(A)	**High consistency:** The best match is a high-confidence identification. The second-best match is (1) a high-confidence identification in which the species is identical to the best match, (2) a low-confidence identification in which the species or genus is identical to the best match, or (3) a non-identification.
(B)	**Low consistency:** The requirements for high consistency are not met. The best match is a high- or low-confidence identification. The second-best match is (1) a high- or low-confidence identification in which the genus is identical to the best match or (2) a non-identification.
(C)	**No consistency:** The requirements for high or low consistency are not met.

Sample 1

**Sample Name:**	F1
**Sample Description:**	
**Sample ID:**	2023-42.1
**Sample Creation Date/Time:**	2023-10-10T15:43:49.825
**Sample Type:**	Standard
**Identification Method:**	MALDI Biotyper MSP Identification Standard Method_MIXED 1.1
**Preprocessing Method:**	MALDI Biotyper Preprocessing Standard Method 1.1
**ACQ Method:**	D:\Methods\flexControlMethods\MBT_FC.par
**AutoXecute Method:**	MBT_AutoX
**Consistency Category (based on 2 best matches):**	A
**Applied Taxonomy Tree:**	Bruker Taxonomy

Rank (Quality)	Matched Pattern	Score Value	NCBI Identifier
1 (+++)	Clostridium innocuum CCUG 46922 CCUG	2.24	1522
2 (+++)	Clostridium innocuum CCUG 36812 CCUG	2.18	1522
3 (+++)	Clostridium innocuum CCUG 45485 CCUG	2.18	1522
4 (+++)	Clostridium innocuum DSM 22910 DSM	2.14	1522
5 (+)	Clostridium innocuum 14162670_3 MVD	1.96	1522
6 (+)	Clostridium innocuum DSM 1286T DSM	1.93	1522
7 (+)	Clostridium innocuum 1079_ATCC 14501T BOG	1.84	1522
8 (+)	Clostridium innocuum HU56448 PNU	1.79	1522
9 (+)	Clostridium innocuum HU46588 PNU	1.74	1522
10 (-)	Clostridium innocuum 15064122_9 MVD	1.46	1522

Sample 2

**Sample Name:**	F2
**Sample Description:**	
**Sample ID:**	2023-42.2
**Sample Creation Date/Time:**	2023-10-10T15:43:49.828
**Sample Type:**	Standard
**Identification Method:**	MALDI Biotyper MSP Identification Standard Method_MIXED 1.1
**Preprocessing Method:**	MALDI Biotyper Preprocessing Standard Method 1.1
**ACQ Method:**	D:\Methods\flexControlMethods\MBT_FC.par
**AutoXecute Method:**	MBT_AutoX
**Consistency Category (based on 2 best matches):**	A
**Applied Taxonomy Tree:**	Bruker Taxonomy

Rank (Quality)	Matched Pattern	Score Value	NCBI Identifier
1 (+++)	Clostridium innocuum CCUG 45485 CCUG	2.23	1522
2 (+++)	Clostridium innocuum CCUG 46922 CCUG	2.12	1522
3 (+++)	Clostridium innocuum DSM 22910 DSM	2.09	1522
4 (+++)	Clostridium innocuum CCUG 36812 CCUG	2.07	1522
5 (+)	Clostridium innocuum DSM 1286T DSM	1.84	1522
6 (+)	Clostridium innocuum 15064122_9 MVD	1.80	1522
7 (+)	Clostridium innocuum 14162670_3 MVD	1.80	1522
8 (+)	Clostridium innocuum 1079_ATCC 14501T BOG	1.78	1522
9 (+)	Clostridium innocuum HU46588 PNU	1.71	1522
10 (-)	Clostridium innocuum HU56448 PNU	1.68	1522
